# Justus' Test for Syphilis

**Published:** 1907-12-21

**Authors:** 


### Points in Diagnosis.

### JUSTUS' TEST FOR SYPHILIS.

It is the function of a medical paper such as The Hospital not merely to bring before practitioners such methods and so forth as are of undoubted value, but also to say something from time to time about the shortcomings of other methods and tests.

Justus' test for syphilis may not be known to all, but as it has the American Army to recommend it, and as it has been employed in some hospitals in this country, it is worth knowing both what it is and what value it has. The test depends upon the changes undergone by the hæmoglobin of the blood after mercurial inunction or mercurial injection. Cabot in his book on the blood gives it in these words: "If, in cases in which secondary symptoms have not yet appeared, we test the hæmoglobin after giving inunction or subcutaneous injection of mercury, we find that within twenty-four hours a very marked fall in hœmoglobin has taken place (10 to 12 per cent.), owing to the action of the mercury on the weakened corpuscles. This sudden fall is followed by a gradual rise, until within a few days the colouring matter is at a point slightly higher than before the mercury was given. In diseases other than syphilis the sudden drop does not occur. After the advent of the secondary symptoms the peculiar reaction of the mercury does not occur." It happens not infrequently that the practitioner would be very glad of a means of determining whether a particular lesion is syphilitic or not, in the early stages, before secondary symptoms appear. Justus' test, if reliable, would be very valuable.

The test has been utilised in a good many cases in the Military Hospital at Curragh Camp, and the results show that it is extremely misleading. Even if one could not exclude syphilis by finding Justus' test negative, it might have been hoped that the occurrence of a positive Justus' test was an indication of syphilis. The test, however, seems to be unreliable both when positive and when negative; that is to say, the patient may give a positive Justus' reaction, and yet develop no indication of syphilis at all; or he may give a negative test and then develop ordinary secondary syphilis a few weeks later. The following are the results obtained at Curragh Camp:

Twenty-two suspected cases of syphilis were tested altogether; of these, nine gave positive Justus' test, and thirteen did not. Of the nine who gave the test, and who therefore ought to have had primary syphilis, only one developed secondaries, the other eight remaining quite well three months later. Of the thirteen who did not give the test, eight developed secondary syphilis, and five remained well, three months later.

In all these cases there was a sore of some kind, so that the net result is that persons with chancroid are even more liable than those with true chancre to give a positive Justus' test, and the test may be negative with either. One batch of patients had the mercury by inunction, the other by injection. It cannot be argued that the mercurial injection or inunction, as the case may be, prevented secondary symptoms appearing in spite of the original sore being a true chancre and not a chancroid; or, at least, even if it is so argued, the result is similar, for nine of the twenty-two cases did develop secondary symptoms, and only in one of these was Justus' test positive.

We can only conclude that the reaction is too unreliable to be utilised in general practice.

